# Telephone treatments in Improving Access to Psychological Therapies services: an analysis of use and impact on treatment uptake

**DOI:** 10.1186/s12888-022-04404-1

**Published:** 2023-02-07

**Authors:** David Saxon, Michael Barkham, Penny Bee, Judith Gellatly, Cintia Faija, Peter Bower

**Affiliations:** 1 Department of Psychology, Clinical and Applied Psychology Unit (CAPU), University of Sheffield, 1 Vicar Lane, S1 2LT Sheffield, UK; 2 School of Health Sciences, Division of Nursing, Midwifery and Social Work, Manchester Academic Health Science Centre, University of Manchester, Manchester, UK; 3 NIHR School for Primary Care Research, Centre for Primary Care and Health Services Research, Manchester Academic Health Science Centre, University of Manchester, Manchester, UK

**Keywords:** Improving Access to Psychological Therapies (IAPT), Telephone Assessment, Telephone Treatment, Attendance, Multilevel modelling, COVID, Patient Case Management Information System (PCMIS)

## Abstract

**Background:**

There is debate about how best to increase access to psychological therapy and deliver mental healthcare effectively and efficiently at a national level. One trend is the increased use of the telephone to deliver therapy. However, there is the potential to disadvantage certain patient groups and/or impact on uptake of help. This study aims to answer three questions: (i) Which factors are associated with being offered an assessment by telephone? (ii) Which factors are associated with attendance at assessment? and (iii) What is the impact of an assessment by telephone on subsequent treatment appointment?

**Methods:**

Routine outcome data was provided by seven UK Improving Access to Psychological Therapy services. The analysis sample comprised 49,923 patients who referred to 615 general practices in 2017. Multilevel modelling, including service and GP practice as random factors, was used to answer the three research questions.

**Results:**

The offer of an initial assessment by telephone was strongly associated with local service configuration. Patient self-referral, a shorter wait, greater age and lower deprivation were associated with attendance at assessment and subsequent treatment session. Telephone mode assessment had no impact on the uptake of the assessment but may influence the uptake of further treatment if this was also by telephone. The practitioner carrying out the assessment had a significant effect on subsequent treatment uptake.

**Conclusion:**

Offering telephone assessments does not have a negative impact on uptake of assessment and services may benefit by facilitating and integrating telephone assessments into their systems. The COVID-19 pandemic has accelerated the use of telephone and other remote means of delivery, and results from this study can inform services to consider how best to re-configure post-pandemic.

**Supplementary Information:**

The online version contains supplementary material available at 10.1186/s12888-022-04404-1.

## Background

Patients with common mental health problems who are living in the community generally prefer psychological therapies to medication [1]. In England, the Improving Access to Psychological Therapies (IAPT) programme was established to increase access to talking treatments for depression and anxiety-related disorders, and promote recovery and work productivity [2]. The programme comprises a stepped care model of service delivery with the majority of treatment-based interventions being low intensity (step 2 care) delivered by a psychological wellbeing practitioner (PWP). These interventions include psycho-education groups, guided self-help (GSH) and computerised CBT (cCBT). Patients referred to the IAPT services are assessed by a PWP and may be treated by them or may be stepped-up to a high intensity treatment, or may be referred-on or signposted to other services. Prior to the COVID pandemic in 2020, PWPs carried out approximately 60% of their non-group sessions by telephone with the remaining 40% by a combination of face-to-face sessions and online support [3].

IAPT reports on data prior to the COVID pandemic indicate that 32.6% of those referred did not receive treatment [4]. Although sizeable, this rate is within the range of those found in similar services (16 − 48%) [5] and comparable with other primary care services in the UK; for example, attendance at general practitioner appointments [6]. Initial attendance and engagement remains an issue in IAPT [7] and as improving access is fundamental to IAPT services, any service delivery developments need to be considered in terms of the barriers to attendance and engagement they may impose or remove for patients, or for particular groups of patients.

With the COVID lockdown and the resulting reconfigurations in services, there has been a large increase in the use of virtual modes of contact, in particular by video. Studies indicate that step 3 therapists and psychiatrists find video sessions largely acceptable, although they highlight computer software problems and accessibility issues [3, 8]. Telephone sessions are accessible to more patients and provide greater ‘anonymity’ for patients and may reduce barriers due to psychological or physical impairment [9] or stigma and sense of shame [10], than face-to-face treatments. National Institute for Health and Care Excellence (NICE) guidelines for mild-to-moderate anxiety and depression [11, 12] include the delivery of psychological interventions delivered by telephone including GSH and computerised cCBT. Although, there may be issues with the quality of the therapeutic alliance with telephone treatment, as there will be fewer non-verbal cues, this may impact more on the therapist, trained in face-to-face treatment, than the patient [13] and a systematic review conducted prior to COVID found no differences in the quality of therapeutic alliance between therapy delivered by telephone or face-to-face [14].

As COVID restrictions ease, IAPT services are likely to reconfigure and provide a range of modes of assessment and treatment for patients including video, in-person face-to-face, and telephone in varying degrees largely dependent on service resources, patient resources, treatment type and preferences. But telephone appointments, for assessments by PWPs in particular, are likely to continue on a large scale due to the combined impact of practicalities, resources, and preferences. Therefore the effect they may have on initial engagement needs to be assessed and possible barriers identified. In the present study of pre-COVID IAPT data of patients referred to a telephone or face-to-face assessment, the aims were to: (i) identify factors associated with an offer of an initial assessment by telephone or face-to-face, (ii) identify factors that are associated with attendance at the initial assessment, and (iii) assess the impact of an assessment by telephone on uptake of the subsequent appointment.

## Method

### Data sample

The data were provided by Patient Case Management Information System (PCMIS), a case management system used by one in three IAPT services across England. PCMIS provides a system for collecting patient data from IAPT services, particularly patient demographics, outcomes and information on the patients’ care pathway, and transferring it into the national IAPT NHS Digital database. In addition to a formal agreement between the research team and PCMIS to use the data in the current study, permissions to analyse their data were also granted by the relevant IAPT services. Ethical approval was granted by the North West-Greater Manchester West Research Ethics Committee (Ref: 18/NW/0372) in 2018 as part of a larger, multi-strand study of telephone interventions in IAPT (the EQUITy Research Programme) which was funded by the National Institute for Health Research (NIHR).

The original dataset comprised data from seven services from 2013 to 2017. Due to large differences between services in their use of telephone appointments (see Supplementary Material, Figs. 1 and 2) and in order to reflect most closely the status of services in 2020, prior to the outbreak of COVID-19 in the UK, we restricted our sample to the most recent years data (2017) giving a sample of 51,191 patients who were offered an assessment either face-to-face or by telephone and were not stepped up to step 3 treatment. Patients who had missing information about treatment mode, or attendance data or had missing or conflicting appointment dates were excluded (*n* = 1268: 2.5%) leaving a study sample of 49,923.

The seven services included were a combination of rural and urban, large and smaller services and when compared to IAPT services nationally on demographic variables were considered broadly representative. In total they contained 615 general practices.

The study sample was predominantly female (60.4%), White (89.9%) and 54.5% of patients came from the two most deprived groups (Index of Multiple Deprivation (IMD) quintile 1, 35.4%; quintile 2, 19.2%) while 13.2% were from the least deprived quintile (quintile 5). IMD quintile is based on the patients’ postcode and is a measure of multiple deprivation in a small geographical area. Although not a true measure, it is often used as a proxy indicator of the deprivation of an individual [15].

As the focus was on initial attendance in treatment, data were limited to the first two patient appointments. Within the data routinely collected by IAPT services [16], variables relevant for the current analysis included: assessment mode (face-to-face or telephone), first treatment session mode (face-to-face, telephone, email, SMS, Talktype or group), whether the patient attended or not, and a number of variables were available as controls in analysis. These comprised: referral source (self-referral or other agency); the “other agency” category was collapsed from 40 sources, predominantly GPs but also a wide range of services and agencies (e.g., Community Mental Health Teams, A&E, Inpatient services, the Voluntary Sector); patient demographic variables (i.e., age, gender, IMD and ethnicity). For ethnicity, categories were collapsed into: White, Black (African and Caribbean), Asian (Indian, Pakistani and Bangladeshi), Mixed Ethnicity and Other. Where the patient was discharged from the service within the first two appointments, the reason for ending was available.

For the analyses of assessment and first treatment session, waiting times between referral date and assessment date and between assessment date and treatment date were calculated. Patient Health Questionnaire-9 (PHQ-9) [17], Generalised Anxiety Disorder-7 (GAD-7) [18], and the Work and Social Adjustment Scale (WSAS) [19] total scores collected at attended assessments were available as measures of depression, anxiety and functioning impairment respectively in the analysis of subsequent treatment attendance.

Missing values on variables were not imputed. For those variables used for service monitoring, few values were missing and imputation would have added significant complexity while the benefits would be marginal. Also, in multilevel data structures and models, particularly with binary outcomes, imputations and multiple imputations of missing values can be unreliable [20].

Two variables had large amounts of missing data which were considered missing not at random as they were generally only collected from patients who attended their assessment. These were employment status and psychological medications use. For employment status, 33.6% were missing overall but for patients who did not attend their assessment the figure was 99.3% compared with 15.4% for those who attended. Similarly for medication use, where the percentages missing were 90.7% and 6.2%, respectively. These two variables therefore were not used in the assessment offer and assessment attendance analysis. However, the sample used for the first treatment appointment analysis was those patients who attended their assessment, therefore the variables were available. For employment status the percentage missing of those that did not attend the treatment appointment was 13.6% compared with 16.7% for those who attended. For medication use the percentages were 4.6% and 4.4%, respectively.

Long-term condition data had 29.9% missing overall, 37.1% of those who did not attend their assessment compared with 27.9% of those who attended. This variable was also included in the analysis of attendance at the treatment appointment and was also assessed in secondary analyses of assessment mode offered and assessment attended.

### Data analysis

Three multilevel logistic regression models were developed to identify variables associated with (i) patients being offered a telephone assessment, (ii) patients attending that assessment, and (iii) patients attending the subsequent first treatment session. Due to inconsistencies in the recording of the purpose of the first two appointments, we considered the initial appointment as the ‘assessment’. The subsequent appointment, if one was offered, was termed the ‘first treatment session’.

Because of the hierarchically, clustered data structure, with potentially four hierarchical levels (i.e., patients, PWPs, general practice, and service), multilevel modelling (MLM) was used [20]. In MLM the higher-level units (PWP, general practice, and service as appropriate) were entered as random factors thereby controlling for clustering effects and providing a measure of the effect on outcome of the variability at each level. This measure, the intra-class correlation coefficient (ICC), is the variance at each level expressed as a proportion of the total variance and is often presented as a percentage.

As the outcome for each analysis is binary, logistic multilevel models were produced using predictive quasi-likelihood (PQL) 2nd order Taylor’s expansion procedures [21] and estimates of higher-level effects used a logistic distribution for the patient level residual variance of 3.29 [20]. MLwiN software version 3.05 was used for developing multilevel models [22].

## Results

We first describe the patients in the study sample and those offered a telephone or face-to-face assessment and present a flow-chart describing the patient pathway from referral to first treatment session. Three analyses are then presented, which identify variables associated with the mode of assessment offered, and the variables associated with attendance at assessment and at the first treatment session. Full models in the form of MLwiN output and including random effects are presented in Supplementary Materials (Figs. 3, 5 and 6).

### Descriptive statistics

Table 1 shows that over twice as many patients were offered a telephone assessment compared to face-to-face (67.8% vs. 32.2%). Patients offered a telephone assessment were, on average, one year younger, 38.9 (SD: 15.0) years compared to 39.9 (SD: 16.0) years, female (68.8% compared with 66.4%) and from average and below average areas of deprivation (IMD Quintiles 3 and 4). Some ethnicity groups were more likely to be offered a telephone assessment (Mixed Ethnicity, 81.7%; Black, 72.6%; and White, 68.8%) while others (Asian, 67.0%; and particularly ‘Other ethnicity’, 58.0%) were more likely to be offered a face-to-face assessment. Most patients self-referred (60.6%) and more of these patients were offered a telephone assessment compared with patients referred from other sources (83.4% vs. 44.4%). Patient descriptives for the samples attending assessment and first treatment session are presented in Supplementary Material (Tables 1 and 2).

**Table 1:** Patient characteristics and the mode of assessment offered

		Mode of Assessment N (valid %)	
	All 49923	Face-to-face 16057 (32.2)	Telephone 33866 (67.8)
Age at referral			
Mean (sd)	39.3 (15.31)	39.9 (15.97)	38.9 (14.98)
Missing	0		
Gender			
Female	31703	9906 (31.2)	21797 (68.8)
Male	17847	5994 (33.6)	11853 (66.4)
Missing	373	157	216
Ethnicity			
White	42384	13205 (31.2)	29179 (68.8)
Mixed Ethnicity	896	164 (18.3)	732 (81.7)
Asian	2758	910 (33.0)	1848 (67.0)
Black	548	150 (27.4)	398 (72.6)
Other ethnicity	566	238 (42.0)	328 (58.0)
Missing	2771	1390	1383
IMD			
Quintile 1 (Most deprived)	17400	6132 (35.2)	11268 (64.8)
Quintile 2	9441	3036 (32.2)	6405 (67.8)
Quintile 3	8466	2043 (24.1)	6423 (75.9)
Quintile 4	7408	2167 (29.3)	5241 (70.7)
Quintile 5 (Least deprived)	6457	2226 (34.5)	4231 (65.5)
Missing	751	453	298
Referral source			
Self	29579	4912 (16.6)	24667 (83.4)
Other	19202	10668 (55.6)	8534 (44.4)
Missing	1142	477	665

### The patient pathway

**Fig. 1:**
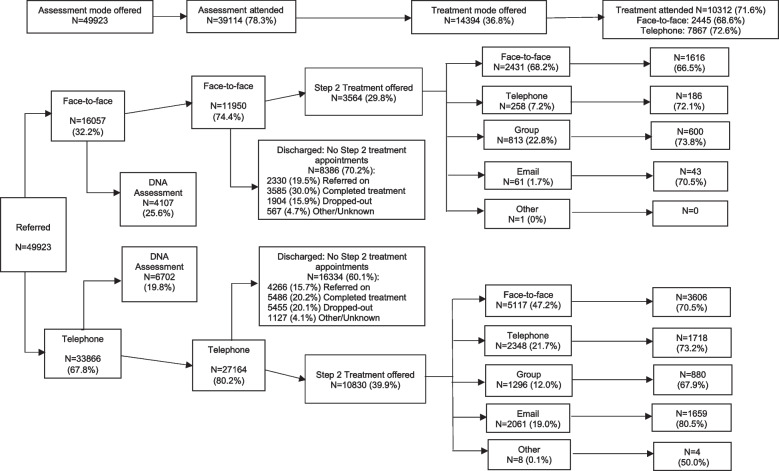
Flowchart of care pathway from referral to first treatment session

Figure 1 describes the patient pathways from referral to the first treatment session. More patients were offered a telephone assessment and more of them attended. Of those offered a telephone assessment 80.2% attended, while 74.4% of those offered a face-to-face assessment attended. A larger percentage of patients were discharged from the service following a face-to-face assessment compared to a telephone assessment (70.2% vs. 60.1%), and these are broken down in Fig. 1 by reason for ending (i.e., referred on, completed treatment, dropped-out, other/unknown). Of all patients offered step 2 treatment sessions (*n* = 14,394), 52.4% were offered face-to-face treatment.

Overall, 10,312 (71.6%) of patients offered treatment attended their first treatment session. Of those who had a telephone assessment (*n* = 10,830), 72.6% attended their first treatment session, while of patients who had a face-to-face assessment (*n* = 3564), 68.6% attended. A telephone assessment followed by a face-to-face treatment session was the most common combination of modes, offered to 35.6% of all patients assessed and offered step 2 treatment.

### Mode of assessment offered

Table 2 lists the patient variables associated with being offered a telephone assessment. Due to missing data on some variables, the model was based on data from 47,730 (95.6%) patients. Table 2 indicates that those offered a telephone assessment were more likely to be self-referrers, of White or Mixed Ethnicity, female, from less deprived areas or younger. Most notably, patients who did not self-refer were around half as likely to be offered a telephone assessment compared to those who self-referred, with an OR (95% CI) of 0.52 (0.47, 0.58).

**Table 2:** Variables associated with being offered a telephone assessment

Variables included	B	SE	OR	OR Lower 95%CI	OR Upper 95%CI	*p*-value
Referral source						
*Reference category*						
Self-referral						
Non Self-Referral	-0.652	0.057	0.52	0.47	0.58	<0.001
Index of Multiple Deprivation (IMD)						
R*eference category*						
Quintile 1						
Quintile 2	0.148	0.043	1.16	1.07	1.26	0.001
Quintile 3	0.244	0.05	1.28	1.16	1.41	<0.001
Quintile 4	0.186	0.054	1.20	1.08	1.34	0.001
Quintile 5	0.219	0.059	1.24	1.11	1.40	<0.001
Gender						
*Reference category*						
Male						
Female	0.148	0.03	1.16	1.09	1.23	<0.001
Ethnicity						
*Reference category*						
White						
Mixed ethnicity	0.074	0.108	1.08	0.87	1.33	0.493
Asian	-0.28	0.064	0.76	0.67	0.86	<0.001
Black	-0.328	0.122	0.72	0.57	0.91	0.007
Other ethnicity	-1.138	0.107	0.32	0.26	0.40	<0.001
Age at Referral-gm	-0.011	0.001	0.99	0.99	0.99	<0.001

The variables identified in Table 2 were produced by a 2-level model (general practice-patient). The general practice effect was estimated at 50.2%, indicating that over half of the variance in the assessment mode offered was due to variability between general practices. There was also a significant random slope for ‘referral source’ indicating that the effect that referral source had on the mode offered varied between practices. (See Supplementary Materials, Fig. 3 for MLwiN output including random effects).

When ‘service’ was included in a 3-level model, the result suggested service might account for around half of the general practice effect. However, its effect estimate had a large standard error, due to the small number of services, and its inclusion had little effect on other variable estimates. Therefore, service was excluded from the model and a 2-level model was used. It is possible that the large general practice effect is split to some degree between practice and service effects, such that we may consider the higher-level effect found here as an ‘organisation effect’.

Long-term condition was added to the model, as a secondary analysis (see Supplementary Material, Fig. 4). It reduced the sample size to 33,969, but showed a significant association with the mode offered, with an OR (95% CI) of 0.83 (0.76, 0.89), indicating patients with a long-term condition were less likely to be offered a telephone assessment. Other variables identified in the primary model remained significant and their effects changed little.

### Patient attendance at assessment

The patient variables associated with attendance at assessment and the impact of assessment mode are presented in Table 3. Patients who were older, from less deprived areas, had self-referred or had a shorter wait were more likely to attend their assessment.

**Table 3:** Variables associated with patient attendance at assessment session

Variables included	B	SE	OR	OR Lower 95%CI	OR Upper 95%CI	*p*-value
Referral source						
*Reference category*						
Self-referral						
Non Self-referral	-0.448	0.037	0.64	0.59	0.69	<0.001
Index of Multiple Deprivation (IMD)						
*Reference category*						
Quintile 1						
Quintile 2	0.207	0.033	1.23	1.15	1.31	<0.001
Quintile 3	0.298	0.037	1.35	1.25	1.45	<0.001
Quintile 4	0.380	0.039	1.46	1.35	1.58	<0.001
Quintile 5	0.413	0.042	1.51	1.39	1.64	<0.001
Age at Referral-gm	0.009	0.001	1.01	1.01	1.01	<0.001
Days between Referral and Assessment-gm	-0.015	0.001	0.99	0.98	0.99	<0.001
Assessment mode offered						
*Reference category*						
Face-to-face						
Telephone	0.03	0.031	1.03	0.97	1.10	0.321

Although a larger percentage of patients offered a telephone assessment attended (Fig. 1), the multivariate analysis indicated that after controlling for other variables (referral source, IMD, waiting time and age), the mode of the assessment had no significant effect on attendance, suggesting any advantage in attendance to telephone assessment is a function of other variables included. Patients from less deprived areas were more likely to attend and were more likely to have been offered a telephone assessment (Table 1). Similarly, most self-referrers (83.4%) were offered a telephone assessment (Table 1) and of those who self-referred, 82.1% attended compared with 72.6% of those referred from other sources.

Patients offered a telephone assessment also had a shorter wait on average. The median (IQR) wait for telephone assessments was 7 (2,18) days compared with 19 (7,33) days for face-to-face assessments; and patients who had a shorter wait were more likely to attend with a median (IQR) wait of 9 (2,21) days for attenders compared with 15 (5,28) days for non-attenders. Long-term condition was not significant in the model.

In this model of assessment attendance, the general practice effect was 5.2%, which was statistically significant but a much smaller effect than for the mode offered. Again, the general practice effect should be considered an ‘organisation effect’ as it includes a small effect of the service.

### Treatment uptake

Of the 14,394 patients offered treatment, those offered Talktype or SMS (*n* = 9) were excluded due to small numbers, as were those whose first treatment session was cancelled by the service (*n* = 653: 6.2% of those were offered a telephone treatment session and 5.9% of those were offered a face-to-face treatment session). Of the remaining sample of 13,732 (10,300 assessed by telephone; 3432 assessed face-to-face), 75.1% of patients attended with an attendance rate for those who had a face-to-face assessment of 71.2%, compared to 76.3% for those who had a telephone assessment.

Table 4 shows that compared to a face-to-face assessment, a telephone assessment increased the likelihood of attending the first treatment session (OR 1.28; 95% CI; 1.10, 1.50). In contrast, the model also shows that patients offered telephone treatment were less likely to attend than patients offered face-to-face treatment (OR 0.76; 95% CI; 0.64, 0.91). A comparison of the model coefficients for telephone indicates that any benefit to treatment session attendance of a telephone assessment is removed where the treatment mode is also telephone. There were no significant interactions between the assessment mode and patient demographic variables or between treatment mode and patient variables indicating that the patient factors had a similar effect on treatment attendance regardless of the treatment session mode.

**Table 4:** Variables associated with patient attendance at the first treatment session

Variables included	B	SE	OR	OR Lower 95%CI	OR Upper 95%CI	*p*-value
Referral source						
*Reference category*						
Self-referral						
Non Self-referral	-0.166	0.058	0.85	0.76	0.95	0.004
Gender						
*Reference category*						
Male						
Female	-0.145	0.055	0.87	0.78	0.96	0.008
Employment status						
*Reference category*						
Employed						
Unemployed seeking work, receiving benefits	-0.185	0.135	0.83	0.63	1.10	0.198
Student	0.478	0.143	1.61	1.24	2.10	<0.001
Long-term sickness	-0.249	0.097	0.78	0.64	0.94	0.01
Homemaker	-0.199	0.107	0.82	0.66	1.01	0.064
Not working, not Seeking work or receiving benefits	-0.209	0.085	0.81	0.69	0.96	0.014
Unpaid voluntary work	-0.763	0.372	0.47	0.22	0.97	0.04
Retired	0.009	0.12	1.01	0.80	1.28	0.94
Index of Multiple Deprivation (IMD)						
*Reference category*						
Quintiles 1-3						
Quintiles 4-5	0.205	0.061	1.23	1.09	1.38	<0.001
Age at Referral-gm	0.018	0.002	1.02	1.01	1.02	<0.001
First Appt. WSAS-gm	-0.008	0.001	0.99	0.99	1.00	0.008
Wait from Appt. 1 to 2(days) -gm	-0.006	0.001	0.99	0.99	1.00	<0.001
Assessment mode						
*Reference category*						
Face-to-face						
Telephone	0.250	0.08	1.28	1.10	1.50	0.002
Treatment mode offered						
*Reference category*						
Face-to-face						
Telephone	-0.269	0.089	0.76	0.64	0.91	0.003
Email	0.364	0.107	1.44	1.17	1.77	0.001
Group	-0.253	0.01	0.78	0.64	0.94	0.012

As an additional analysis, a model which included an eight-category variable combining the mode of the assessment and the mode of the treatment session was produced. This indicated that compared to the most common combination, telephone assessment and face-to-face treatment, patients who had a telephone assessment and telephone treatment (OR 0.67 (0.56, 0.81) or a face-to-face assessment and face-to-face treatment (OR 0.64; 95% CI; 0.53, 0.77) were both less likely to attend a first treatment session (see Supplementary Material, Table 3).

Regarding the control variables in Table 4, preliminary analysis found that IMD quintiles 2 and 3 were not significantly different to quintile 1 in their association with attendance at first treatment session; therefore, IMD was collapsed into two categories, quintiles 1 to 3 and quintiles 4 to 5. Employment status, psychological medication use and long-term conditions were also considered in this model.

Patients who self-referred, had shorter waits, were male, less deprived, older, or had better functioning were more likely to attend. Employment status was included in this model and showed that compared to employed patients, students were more likely to attend while patients who had a long-term sickness, or were not seeking work or were in unpaid voluntary work, were less likely to attend. Long-term condition and psychological medication use were not significant in the model. Also, severity of depression and anxiety, as measured by the PHQ-9 and GAD-7 at the assessment session were not associated with attending their first treatment session.

The model was based on 9540 (69.5%) of the 13,732 patients in the sample which was largely due to missing values on WSAS and employment status. There was no significant interaction between WSAS and gender but excluding WSAS from the model resulted in gender becoming non-significant, suggesting some relationship between gender and level of functioning in their association with first session treatment attendance. A model excluding both WSAS and employment status (*N* = 13,429) shows similar effects for remaining variables included. (See Supplementary Material, Fig. 7).

A comparison of waiting times found that patients offered telephone treatment had a median (IQR) wait of 24 (14,38) days compared with 48 (28,76) days for face-to-face treatment. Email and group treatments had waits of 35 (28,44) days and 33 (20,47) days, respectively.

The PWP who conducted the assessment was included as a variable in the model of first treatment session attendance and a significant PWP effect of 11.5% was found. Therefore, after controlling for other variables (referral source, gender, IMD, age, WSAS score, waiting time, employment status and the modes of the assessment and the treatment sessions), over 10% of the variance in patient attendance at the first treatment session was associated with the PWP seen at the assessment. There were no significant random slopes for assessment mode or treatment mode variables in the model, indicating that the PWP effect on patient attendance at first treatment session is similar regardless of the mode of the assessment or the mode of the treatment. The service and general practice effects were also tested in the model, but both effects were small and not significant (approximately 1.4% and 0.4%, respectively) and both were excluded from the model.

## Discussion

In order to improve access to psychological therapies and meet growing demand and targets set by IAPT, the use of telephone in IAPT services has increased over time. In this large-scale study of pre-COVID pandemic routinely collected IAPT data, we aimed to identify those patients most likely to be offered a telephone assessment and the impact it may have on assessment attendance and subsequent treatment attendance. The results will help services make evidence-based informed decisions about delivery post-pandemic. Even with the increase in the use of video and other virtual technologies during COVID and since, it is likely that telephone assessments will continue to comprise a large number of assessments due to their familiarity for PWPs and patients and existing techno logical systems at services. New technologies may be available for treatment, but telephone treatment will remain an important option due to greater accessibility and particularly for those step 2 treatments requiring only telephone support.

The large organisation effect (50%) found for the mode of assessments offered is a likely indicator of the extent of variability in options available to different services, determined by local resources and culture; for example, the availability of rooms or telephones and staff attitudes to the relative merits of each contact mode. The organisation had a much smaller significant effect (5%) on assessment attendance and a non-significant effect on first treatment session attendance.

Controlling for the large organisation effect, patients who self-referred were younger, female, from less socially and economically deprived areas, or White or Mixed Ethnicity were most likely to be offered telephone assessments. This is broadly consistent with findings in the literature for patients considered less difficult to engage [23, 24]. We can only speculate as to why females and younger patients were more likely to be offered telephone assessments and patients with long-term conditions were more likely to be offered a face-to-face assessment. It may be the perception of services that telephone appointments would be more accessible to females due to the greater time demands (i.e., family/caring responsibilities additional to employment demands). Similarly, offering telephone assessments to younger patients may reflect a conscious or unconscious effort by services to better engage younger patients believing them to be more at ease and adaptive to remote communications media such as telephones. For patients with a long-term condition it may be important for the PWP to assess their condition, face-to-face.

We found that the mode of the assessment in itself was not associated with assessment attendance, and patients more likely to attend were those who were older or from less deprived areas or had self-referred or had a shorter wait. These groups of patients and those with less impaired functioning were also more likely to attend treatment independently of mode of first treatment session. In line with other studies [25], younger patients were less likely to attend both assessment and treatment, while gender was not associated with assessment attendance, and males were more likely to attend the first treatment session.

Both the mode of assessment and the mode of treatment offered were associated with attendance at treatment. A telephone assessment was associated with higher attendance at face-to-face and email treatment but had no advantage over a face-to-face assessment when the treatment was telephone or group. This suggests that although patients found a telephone assessment acceptable, perhaps because it was more common practice and/or they entered the service and were assessed more quickly, they felt telephone treatment was less acceptable, often despite a shorter wait. Also, patients who had a telephone assessment were less likely to attend a first treatment session by telephone compared to face-to-face suggesting that patients had a more negative view of telephone treatment if they had attended a telephone assessment than if they attended a face-to-face assessment.

Telephone treatment was less familiar to patients prior to COVID and it may not have met some patients’ expectations of what therapy should be [26], although we found patients were more likely to attend treatment via email than either telephone and face-to-face treatment. The reason for this is difficult to assess. It may in part reflect a reduced inconvenience or burden for patients, greater anonymity and less stigma even compared to telephone treatment [13, 27, 28] and/or the ease at which treatments such as GSH or cCBT can be administered by email.

The impact that the PWP carrying out the assessment had on subsequent treatment attendance (11% of the variance) is comparable to studies of more intensive therapy drop-out [29, 30]. Some PWPs at assessment were better able to facilitate a patient’s future attendance, regardless of mode of the assessment and the treatment. Further research is required to identify what might be driving this effect. Research has indicated that negative beliefs, lack of telephone skills training and limited knowledge for the rationale of services using telephone modality could interfere/impact on practitioners' acceptance/uptake of telephone use [31].

As services may be expanding their use of telephone assessments and treatment even further after the pandemic, this study provides guidance and insight into the effects this may have on the attendance of different patient groups and which groups may require additional support in order to engage with the telephone mode. Increasing facilities and systems for self-referral and reducing waiting times should improve attendance, particularly at assessment, and the adoption of telephone assessments can achieve both. Services should also consider how the assessments by PWPs might be improved to become more consistent and increase subsequent attendance. The need for practitioner training has been highlighted in studies of video treatments [3].

It is important to highlight that telephone is a mode to deliver assessment and psychological treatment and patient suitability for its use should be addressed. Patient choice may increase with additional treatment modes becoming available, however, some evidence has revealed tensions between the political ideology of patient choice and practical service delivery constraints, indicating modality is usually a function of service design rather than of patient choice [32]. Patients should be offered a choice to receive assessment/treatment using different modalities and waiting times should not be different across modes.

### Strengths and limitations

To our knowledge, this is the first study to use a very large multi-site sample of routinely collected service data to assess the effect of telephone assessments on treatment attendance in real-world practice compared with face-to-face assessments and treatment, controlling for other significant variables. In addition, the analysis included and accounted for the variability between services and between practitioners.

It is a limitation that large-scale data collected since the onset of the COVID pandemic were not available for this study, which was designed and conducted prior to the pandemic following timelines for a NIHR research programme. Within those timelines it was not possible to apply for additional data, obtain permissions from services, prepare and conduct analysis. However, with the likelihood that telephone sessions will continue to be an important mode for PWP assessments and treatments, possible barriers and benefits found in pre-COVID data will still be relevant. Also, recent published studies that have reported on data collected during COVID restrictions have focussed on, for example, high intensity treatments [3] or specific patient sub-groups [33, 34] or they have not provided enough details regarding the modes of delivery [35–38].

As it is currently unclear how IAPT services will reconfigure as they move forward following the reduction in COVID restrictions, this study can inform that reconfiguration by identifying patient subgroups whose access to services may be disadvantaged by a reduction in in-person, face-to-face provision. Also, the study provides a pre-pandemic ‘baseline’ which can inform future research, particularly the findings of significant organisation and practitioner effects. Future large-scale studies should consider how the range of modes currently available may impact on attendance.

Although the size of the dataset is a strength, routinely collected data also imposes limitations on the study. Some potentially important variables may have large amounts of missing data or may not be available at all. Where possible, models were produced with and without variables with large numbers of missing values and the differences are reported. However, it is possible that variables not available may be confounding the associations reported. For example, the association of ethnicity group with assessment mode offered may be confounded by the need for an interpreter, data for which were not available. Also, employment status was not available in the analysis of assessment mode offered and assessment attendance, but it was found to be significant in attendance at first treatment session and it is possible that it would also be associated with assessment mode offered and attended which may confound the associations of the other significant variables found. However, most of the patient variables often associated with attendance were included and the large sample size allows for reliable estimates of the available variable associations.

In addition, the number of services was limited to 7 which may have prevented the development of more complex models as well as more accurate and reliable estimates of the service effects. Ideally, more than 50 services and possibly over 100 would be required [39]. Although not ideal, in the current study we considered the effect of the service and the GP practice as a combined ‘organisation effect’.

## Conclusion

As psychological treatment services recover from the impact of COVID, they will face complex decisions about reconfiguring services, and whether innovations introduced in the pandemic should endure. Telephone sessions will continue to be an important option and our analyses suggest that offering telephone assessments does not have a negative impact on attendance at assessment and services may benefit by facilitating and integrating telephone assessments into their systems. Telephone treatment appears less acceptable to patients than face-to-face treatment, while the practitioner carrying out the assessment was more important for attendance at both modes of treatment. Future research of step 2 data collected during the past three years may provide insight into the acceptability of telephone treatment when in person, face-to-face alternatives were not possible and other modes such as video have become available.

## Supplementary Information

**Additional file 1:** **Table 1.** Descriptives of patients attending assessment session. **Table 2.** Descriptives of patients offered the first treatment session. **Table 3.** Variables associated with attendance at first treatment session including the combinations of assessment and treatment session modes as 8 categories. **Figure 1.** Proportion of telephone assessments offered by services by year. **Figure 2.** Proportion of telephone assessments offered by services broken down by IAPT service A-G, (*N*=7). **Figure 3.** Model of patients offered a telephone assessment. **Figure 4.** Model  of patients offered a telephone assessment with long-term condition included. **Figure 5.** Model of patient attendance at assessment. **Figure 6.** Model of patient attending their first treatment session. **Figure 7.** Model of patients attending their first treatment session with WSAS and Employment status excluded

## Data Availability

The data that support the findings of this study are available from [Patient Case Management Information System (PCMIS),] but restrictions apply to the availability of these data, which were used under license for the current study, and so are not publicly available. Data are however available from the authors upon reasonable request and with permission of PCMIS.

## Abbreviations

cCBT: Computerised CBT
CI: Confidence Interval
GAD-7: Generalised Anxiety Disorder-7
GM: Grand mean
GSH: Guided Self-Help
HSCIC: Health and Social Care Information Centre
IAPT: Improving Access to Psychological Therapies
ICC: Intra-class correlation coefficient
IMD: Index of Multiple Deprivation
IQR: Interquartile range
MLM: Multilevel modelling
NICE: National Institute for Health and Care Excellence
OR: Odds Ratio
PCMIS: Patient Case Management Information System
PHQ-9: Patient Health Questionnare-9
PQL: Predictive quasi-likelihood
PWP: Psychological Wellbeing Practitioner
SD: Standard Deviation
SMS: Short Message Service
WSAS: Work and Social Adjustment Scale
