# Supplementary material for: Telephone treatments in Improving Access to Psychological Therapies services: an analysis of use and impact on treatment uptake
Source: BMC Psychiatry. 2023 Feb 7;23:95. doi: 10.1186/s12888-022-04404-1 (PMC9903253; doi:10.1186/s12888-022-04404-1)

**Supplementary Material**

**Table 1.** Descriptives of patients attending assessment session

|  |  | Assessment mode | |
| --- | --- | --- | --- |
|  | All  39114 | Face-to-face  11950 | Telephone  27164 |
| **Age at referral:**  Mean (SD) | 39.7 (15.43) | 40.7 (16.24) | 39.2 (15.04) |
| **IMD:**  Quintile 1 n(%)  Quintile 2 n(%)  Quintile 3 n(%)  Quintile 4 n(%)  Quintile 5 n(%)  Missing | 12926  7436  6845  6058  5279  570 | 4286 (33.2)  2260 (30.4)  1568 (22.9)  1717 (28.3)  1762 (33.4)  357 | 8640 (66.8)  5176 (69.6)  5277 (77.1)  4341(71.7)  3517 (66.6)  213 |
| **Referral source:**  Self n(%)  Other n(%)  Missing | 24298  13936  880 | 3900 (16.1)  7661 (55.0)  389 | 20398 (83.9)  6275 (45.0)  491 |
| **Waiting time in days**  Referral to Assessment. Mean (SD)  Missing | 13.9 (14.66)  12 | 20.3 (17.91)  5 | 11.1 (11.93)  7 |

**Table 2.** Descriptives of patients offered the first treatment session

|  |  | Mode of treatment session | | | |
| --- | --- | --- | --- | --- | --- |
|  | All  13732^1^ | Face-to-face  7102 | Telephone  2444 | Email  2101 | Group  2085 |
| **Age at referral:**  Mean (SD) | 39.9  (15.52) | 39.9  (15.38) | 41.1  (16.24) | 37.3  (13.80) | 41.2  (15.75) |
| **Gender:**  Female  Male  Missing | 8690  5007  35 | 4484 (51.6)  2598 (51.9)  20 | 1562 (18.0)  877 (17.5)  5 | 1360 (15.7)  739 (14.8)  2 | 1284 (14.8)  793 (15.8)  8 |
| **Ethnicity:**  White  Mixed Ethnicity  Asian  Black  Other ethnicity  Missing | 12012  245  777  154  137  407 | 6115 (50.9)  123 (50.2)  513 (66.0)  70 (45.5)  78 (56.9)  203 | 2127 (17.7)  57 (23.3)  133 (17.1)  33 (21.4)  26 (19.0)  68 | 1921 (16.0)  33 (13.5)  46 (5.9)  21 (13.6)  8 (5.8)  72 | 1849 (15.4)  32 (13.1)  85 (10.9)  30 (19.5)  25 (18.2)  64 |
| **Employment status:**  Employed  Unemployed, seeking work & receiving benefits  Student  Long-term sickness  Homemaker  Not working, not Seeking work or receiving benefits  Unpaid Voluntary work  Retired  Missing | 6712  372  534  996  649  1166  44  1068  2191 | 3552 (52.9)  208 (55.9)  297 (55.6)  652 (65.5)  321 (49.5)  767 (65.8)  15 (34.1)  561 (52.5)  729 | 841 (12.5)  50 (13.4)  80 (15.0)  150 (15.1)  73 (11.2)  154 (13.2)  10 (22.7)  199 (18.6)  887 | 1317 (19.0)  69 (18.5)  77 (14.4)  142 (21.9)  142 (21.9)  107 (9.2)  13 (29.5)  98 (9.2)  191 | 1002 (14.9)  45 (12.1)  80 (15.0)  107 (10.7)  113 (17.4)  138 (11.8)  6 (13.6)  210 (19.7)  384 |
| **IMD:**  Quintile 1  Quintile 2  Quintile 3  Quintile 4  Quintile 5  Missing | 4165  2592  2579  2308  1969  119 | 2593 (62.3)  1459 (56.3)  1223 (47.4)  969 (42.0)  790 (40.1)  68 | 810 (19.4)  455 (17.6)  430 (16.7)  415 (18.0)  325 (16.5)  9 | 202 (4.8)  316 (12.2)  554 (21.5)  554 (24.0)  457 (23.2)  18 | 560 (13.4)  362 (14.0)  372 (14.4)  370 (16.0)  397 (20.2)  24 |
| **Referral source:**  Self  Other  Missing | 9180  4365  187 | 4283 (46.7)  2661 (61.0)  158 | 1873 (20.4)  560 (12.8)  11 | 1729 (18.8)  371 (8.5)  1 | 1295 (14.1)  773 (17.7)  17 |
| **Waiting time in days**  Referral to Assessment. Mean (SD)  Missing  Assessment to Treatment. Mean (SD)  Missing | 12.5 (13.83)  4  44.8 (31.35)  0 | 13.2  (15.30)  0  54.8  (34.85) | 8.7  (11.95)  3  30.1  (25.51) | 11.6  (8.68)  0  36.5  (15.88) | 15.7  (13.74)  1  36.3  (24.11) |
| **WSAS at Assessment**  Mean (SD)  Missing | 19.0 (10.05)  3140 | 19.7 (10.57)  1658 | 19.1 (9.81)  1026 | 18.1 (8.91)  82 | 17.6 (9.64)  374 |

^1^Treatment appointments cancelled by the service (N=653) or SMS or Talktype offered (N=9) were excluded from the sample of 14394

**Table 3.** Variables associated with attendance at first treatment session including the combinations of assessment and treatment session modes as 8 categories

|  | B | SE | OR | Lower  95%CI | Upper  95%CI | p-value |
| --- | --- | --- | --- | --- | --- | --- |
| Fixed Part |  |  |  |  |  |  |
| cons | 1.654 | 0.075 | 5.23 | 4.51 | 6.06 | 0.001 |
| *Reference Category: Self* |  |  |  |  |  |  |
| Non Self_REF | -0.191 | 0.058 | 0.83 | 0.74 | 0.93 | 0.001 |
|  |  |  |  |  |  |  |
| *Reference IMD quintiles1-3* |  |  |  |  |  |  |
| IMD quintiles 4 & 5 | 0.206 | 0.058 | 1.23 | 1.10 | 1.38 | <0.001 |
|  |  |  |  |  |  |  |
| *Reference category: Male* |  |  |  |  |  |  |
| Female | -0.13 | 0.052 | 0.88 | 0.79 | 0.97 | 0.012 |
|  |  |  |  |  |  |  |
| Days between Sessions 1&2-gm | -0.007 | 0.001 | 0.99 | 0.99 | 0.99 | <0.001 |
| Assessment WSAS total-gm | -0.01 | 0.003 | 0.99 | 0.98 | 1.00 | <0.001 |
| Age At Referral-gm | 0.016 | 0.002 | 1.02 | 1.01 | 1.02 | <0.001 |
|  |  |  |  |  |  |  |
| Assessment and treatment sessions modes combined  *Reference category: Phone_F2F* |  |  |  |  |  |  |
| F2F_F2F | -0.448 | 0.093 | 0.64 | 0.53 | 0.77 | <0.001 |
| Phone_Phone | -0.394 | 0.094 | 0.67 | 0.56 | 0.81 | <0.001 |
| F2F_Email | -0.241 | 0.353 | 0.79 | 0.39 | 1.57 | 0.494 |
| Phone_Email | 0.229 | 0.103 | 1.26 | 1.03 | 1.54 | 0.027 |
| F2F_Phone | -0.363 | 0.203 | 0.70 | 0.47 | 1.04 | 0.073 |
| F2F_Group | -0.344 | 0.136 | 0.71 | 0.54 | 0.93 | 0.011 |
| Phone_Group | -0.491 | 0.12 | 0.61 | 0.48 | 0.77 | <0.001 |
|  |  |  |  |  |  |  |
| Random Part |  |  |  |  |  |  |
| Level: PWP |  |  |  |  |  |  |
| Var(cons) | 0.409 | 0.055 |  |  |  |  |
| Level: Patient |  |  |  |  |  |  |
| Var(bcons.1) | 1 | 0 |  |  |  |  |
|  |  |  |  |  |  |  |
| PWPs N | 744 |  |  |  |  |  |
| Patients N | 10339 |  |  |  |  |  |

F2F: Face-to-face treatment. The combinations of assessment and treatment modes are in the format ‘Assessment session mode_Treatment session mode’

**Figure 1.** Proportion of telephone assessments offered by services by year

**Figure 2.** Proportion of telephone assessments offered by services broken down by IAPT service A-G, (N=7)

**Figure 3.** Model of patients offered a telephone assessment


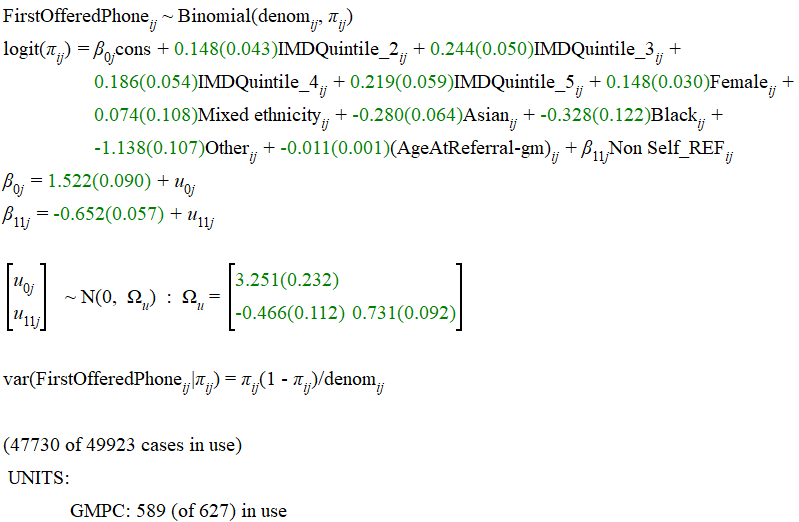


**Figure 4.** Model of patients offered a telephone assessment with long-term condition included


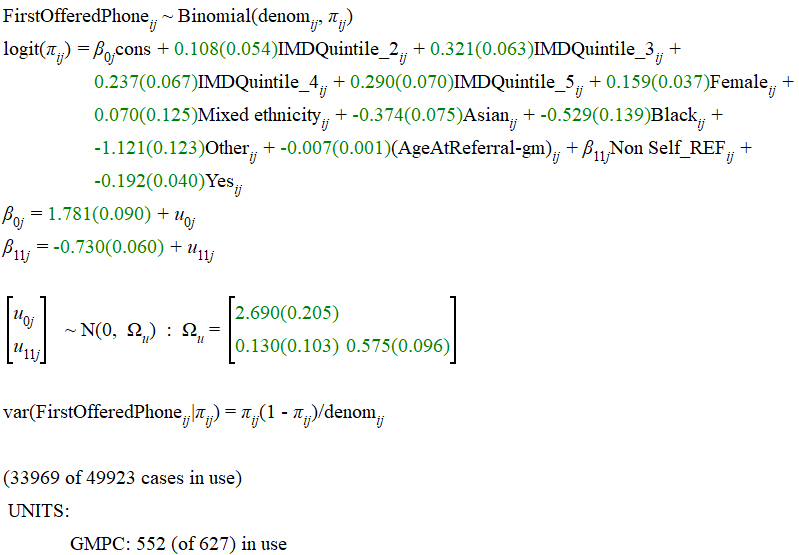


**Figure 5.** Model of patient attendance at assessment


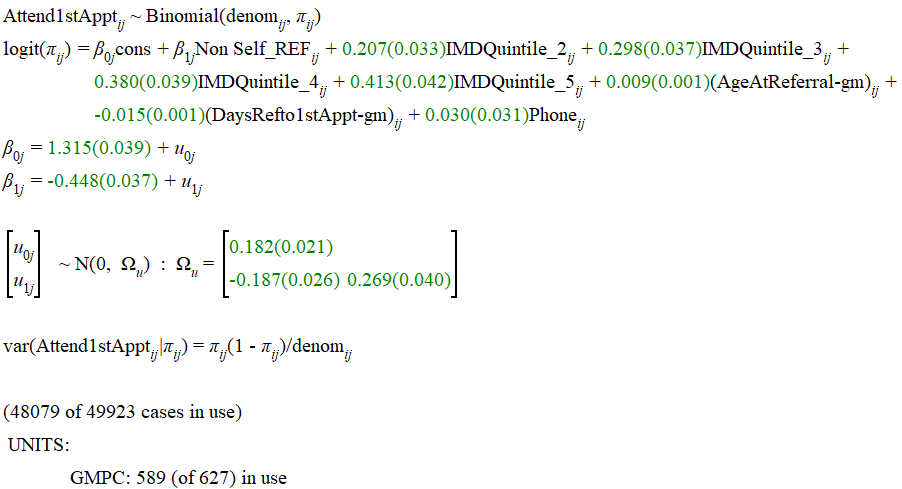


**Figure 6.** Model of patient attending their first treatment session


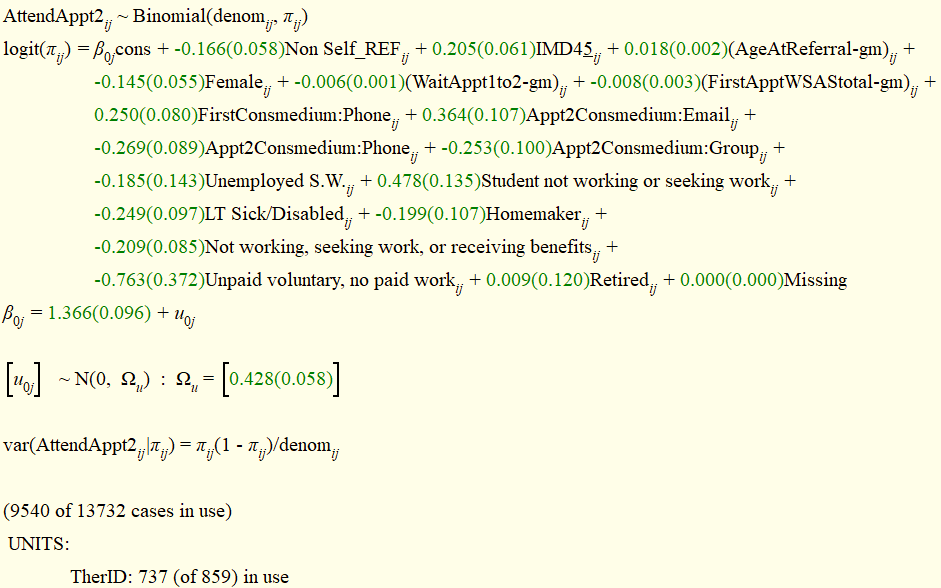


**Figure 7.** Model of patients attending their first treatment session with WSAS and Employment status excluded


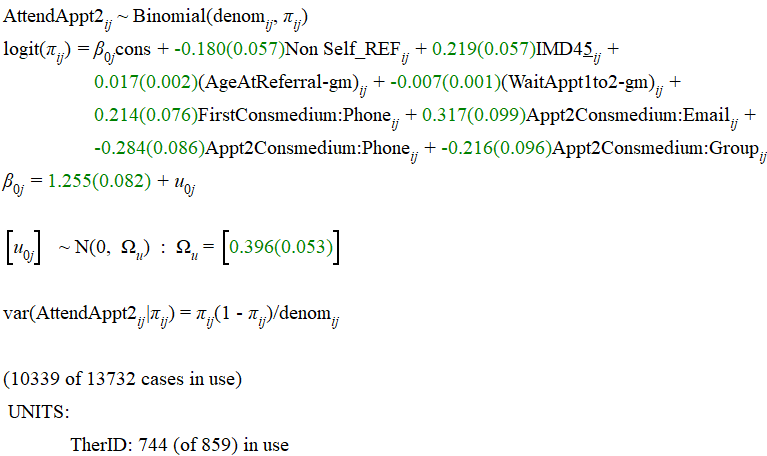

Supplement: Supplementary file 1 — Additional file 1: Table 1. Descriptives of patients attending assessment session. Table 2. Descriptives of patients offered the first treatment session. Table 3. Variables associated with attendance at first treatment session including the combinations of assessment and treatment session modes as 8 categories. Figure 1. Proportion of telephone assessments offered by services by year. Figure 2. Proportion of telephone assessments offered by services broken down by IAPT service A-G, (N=7). Figure 3. Model of patients offered a telephone assessment. Figure 4. Model of patients offered a telephone assessment with long-term condition included. Figure 5. Model of patient attendance at assessment. Figure 6. Model of patient attending their first treatment session. Figure 7. Model of patients attending their first treatment session with WSAS and Employment status excluded [file 12888_2022_4404_MOESM1_ESM.docx]
